# Molecular Dynamics Analysis of the Stereoselective Recognition of Myo-Inositol and D-Chiro-Inositol in a Protein-Based Biosensor

**DOI:** 10.3390/s26123765

**Published:** 2026-06-12

**Authors:** Flavio Rizzo, Enrico De Smaele, Andrea M. Isidori

**Affiliations:** Department of Experimental Medicine, Sapienza University of Rome, Viale Regina Elena 324, 00161 Rome, Italy; enrico.desmaele@uniroma1.it (E.D.S.); andrea.isidori@uniroma1.it (A.M.I.)

**Keywords:** myo-inositol, D-chiro-inositol, biosensor selectivity, molecular dynamics, protein–ligand interactions, stereochemical recognition, computational modeling

## Abstract

**Highlights:**

**What are the main findings?**

- Molecular dynamics simulations reveal distinct protein–ligand interaction networks for myo-inositol and D-chiro-inositol.
- Myo-inositol induces persistent hydrogen bonding and local protein stabilization, unlike its stereoisomer.

**What are the implications of the main findings?**

- Biosensor selectivity originates from ligand-induced protein dynamics rather than static binding affinity.
- Computational analysis provides a mechanistic framework for rational biosensor design toward stereochemically similar analytes.

**Abstract:**

The selective detection of small, highly hydrophilic metabolites differing only in stereochemistry represents a major challenge in biosensor development. Here, we present a computational investigation to elucidate the molecular basis of the experimentally observed selectivity of a protein-based electrochemical biosensor toward myo-inositol over D-chiro-inositol. Although the two stereoisomers differ only in the orientation of a single hydroxyl group, they induce distinct dynamic effects on the protein recognition element. Molecular docking revealed comparable binding regions and similar affinity scores, indicating that selectivity does not arise from differences in binding site or docking energy. To investigate dynamic contributions, all-atom molecular dynamics simulations were performed in triplicate (3 × 100 ns) using the AMBER99SB force field and explicit TIP3P water. Trajectory analyses showed that myo-inositol forms a more persistent hydrogen bond network, resulting in reduced residue-level flexibility, more stable ligand–protein interactions, and enhanced local structural stabilization. Overall, these findings support a dynamic model of stereoselective recognition in which ligand-induced modulation of protein conformational ensembles, rather than static affinity, governs biosensor performance. This work highlights the value of molecular dynamics simulations in the rational design of biosensors targeting structurally similar analytes.

## 1. Introduction

Inositols are a family of naturally occurring cyclohexanehexols that play central roles in eukaryotic physiology through their involvement in membrane biogenesis, intracellular signaling, and metabolic regulation. Within the inositol phosphate/phosphoinositide network, these small polyhydroxylated metabolites act as structural precursors and second messenger components governing calcium mobilization, vesicular trafficking, cytoskeletal rearrangement, and insulin-mediated pathways [1,2,3]. Among the nine possible stereoisomers, myo-inositol is by far the most abundant form in mammalian cells and represents the biochemical backbone of phosphatidylinositol and its phosphorylated derivatives. In contrast, D-chiro-inositol, although less prevalent, fulfills important roles in insulin signaling and glucose homeostasis, and alterations in the physiological myo-/D-chiro-inositol ratio have been associated with metabolic disorders including insulin resistance and polycystic ovary syndrome [4,5,6].

Remarkably, myo-inositol and D-chiro-inositol differ only in the stereochemical orientation of a single hydroxyl group. From a chemical standpoint, this subtle configurational change does not alter molecular formula, size, or overall polarity. However, from a molecular recognition perspective, stereochemistry can dramatically influence hydrogen bond geometry, spatial complementarity, solvation dynamics, and entropic contributions to binding [7]. In protein-based recognition systems, especially those relying on networks of polar residues, even minimal stereochemical variations may propagate into significant differences in interaction cooperativity and persistence.

The analytical discrimination of stereoisomers that differ only by hydroxyl orientation represents a substantial challenge. Conventional electrochemical detection strategies often rely on redox-active species or catalytic amplification, neither of which is intrinsically selective for stereochemical configuration. When dealing with highly hydrophilic and redox-inactive metabolites such as inositols, selectivity must instead arise from the molecular recognition properties of the biofunctional layer immobilized at the electrode interface. Protein-modified electrodes are particularly attractive in this regard, as they combine structural specificity with the capability to transduce binding events into measurable electrochemical signals [8,9,10]. In this context, computational approaches can contribute to the rational engineering of bioreceptor systems by identifying interaction patterns, dynamic stabilization effects, and ligand-dependent conformational responses that may influence biosensor selectivity and signal generation. Understanding how subtle stereochemical differences modulate the behavior of immobilized recognition elements is therefore important for the development of biosensors targeting structurally similar analytes.

Recently, a stereoselective voltammetric biosensor for myo-inositol based on a protein-functionalized electrode surface was reported, showing a significantly stronger electrochemical response toward myo-inositol compared to its stereoisomer D-chiro-inositol [11]. In that study, preliminary computational analyses, including molecular docking, were performed to identify plausible binding regions and interaction modes. However, the previous computational analysis was limited to static docking-derived interaction models and did not investigate the dynamic behavior of the protein–ligand complexes over time.

Building upon those findings, the present work extends the computational investigation by applying all-atom molecular dynamics simulations to the docking-derived complexes, with the aim of elucidating the dynamic determinants of stereoselective recognition.

Although the two molecules differ only in the orientation of a single hydroxyl group, the biosensor exhibited a clear discrimination between the two analytes. The molecular basis of this stereoselective recognition, however, remains unclear.

Computational modeling offers a complementary and mechanistically informative approach to investigate such phenomena. Molecular docking can generate plausible binding hypotheses based on geometric complementarity, but static docking scores alone are often insufficient to capture the subtleties of stereochemical discrimination, particularly for small, flexible, and highly solvated ligands. All-atom molecular dynamics (MD) simulations enable the characterization of time-dependent properties such as hydrogen bond lifetimes, solvent-mediated competition, conformational plasticity, and ligand-induced modulation of protein flexibility [12,13,14,15].

Recent advances in biomolecular simulations have emphasized that molecular recognition often arises from differences in conformational ensembles rather than from static binding affinity alone. In this dynamic view of protein–ligand recognition, ligands may influence the distribution of accessible conformational states of the protein scaffold, thereby modulating interaction stability and functional behavior [16,17].

In this work, we investigate the molecular origin of the stereoselective response observed in a protein-based myo-inositol biosensor through molecular docking and molecular dynamics simulations. By comparing the dynamic behavior of the protein in a complex with myo-inositol and its stereoisomer D-chiro-inositol, we aim to determine whether biosensor selectivity arises from differences in ligand-induced protein dynamics rather than from static binding affinity alone. Starting from docking-derived binding poses, we performed extensive all-atom molecular dynamics simulations of the two protein–ligand complexes and analyzed global structural stability, residue-level flexibility, ligand–protein distances, contact persistence, and secondary structure evolution. The aim of this study is to elucidate how subtle stereochemical differences between the two ligands influence protein interaction patterns and dynamic behavior, thereby contributing to the stereoselective sensing response observed experimentally.

## 2. Materials and Methods

### 2.1. Ligand and Protein Preparation

The three-dimensional structures of myo-inositol and D-chiro-inositol were generated in their neutral forms, consistent with physiological pH conditions (7.4), where all hydroxyl groups remain protonated. Particular attention was devoted to the correct assignment of stereochemistry at each chiral center, as the two ligands differ exclusively in the spatial orientation of a single hydroxyl group, a feature expected to influence hydrogen bond geometry and interaction patterns within the protein recognition region.

Initial ligand geometries were subjected to energy minimization using molecular mechanics in order to remove unfavorable torsional conformations prior to docking and molecular dynamics simulations.

Ligand parameters and topology files compatible with the AMBER force-field framework were generated using ACPYPE (AnteChamber PYthon Parser interfacE, version: 2022.7.21; University of São Paulo, Brazil) [18], which automatically converts parameters derived from the Antechamber tool into formats suitable for molecular dynamics simulations.

The protein recognition element used in the biosensor system was prepared starting from its experimentally determined three-dimensional structure. Hydrogen atoms were added according to standard protonation states at physiological pH. Missing side chains were reconstructed when necessary using backbone-dependent rotamer libraries. The structure was then subjected to restrained energy minimization to remove steric clashes while preserving the experimentally resolved backbone conformation.

### 2.2. Molecular Docking

Docking simulations were performed building upon previously identified binding regions and interaction hypotheses reported in our earlier work [11], where the same protein-based biosensor system was experimentally and computationally characterized.

Molecular docking simulations were performed to generate initial binding configurations of myo-inositol and D-chiro-inositol within the protein recognition region.

Although this approximation does not account for full protein conformational flexibility during ligand recognition, docking was employed here primarily to generate plausible initial binding poses for subsequent molecular dynamics simulations, which provide a more comprehensive description of protein–ligand dynamics in explicit solvent conditions [19].

The docking search space was defined around the experimentally relevant binding region of the protein. Multiple independent docking runs were performed for each stereoisomer in order to ensure adequate conformational sampling.

The resulting docking poses were ranked according to the scoring function implemented in the docking software. High-ranking conformations were evaluated based on hydrogen bond geometry, steric compatibility with surrounding residues, and overall interaction plausibility. Representative poses exhibiting favorable hydrogen bond networks were selected as starting configurations for molecular dynamics simulations.

### 2.3. Molecular Dynamics Simulations

All-atom molecular dynamics simulations were performed using the GROMACS simulation package (version 2026.0; GROMACS Development Team, Uppsala University, Sweden, and the University of Groningen, The Netherlands) [20,21].

Two systems were investigated:

1. The protein–myo-inositol complex;
2. The protein–D-chiro-inositol complex.

For each system, three independent simulations (triplicates) of 100 ns were carried out, resulting in a total simulated time of 300 ns per complex. Independent simulations were initiated using different random velocity seeds to improve statistical sampling.

The AMBER99SB force field was used to describe the protein [22], while ligand parameters generated via ACPYPE were fully compatible with the AMBER framework.

Each system was solvated in an explicit water box using the TIP3P water model [23], and counterions were added to neutralize the total system charge.

Energy minimization was performed using the steepest descent algorithm until convergence of the maximum force criterion.

Equilibration was carried out in two stages:

1. NVT (constant Number of particles, Volume, and Temperature) ensemble equilibration, allowing stabilization of the system temperature;
2. NPT (constant Number of particles, Pressure, and Temperature) ensemble equilibration, allowing stabilization of pressure and solvent density.

Temperature was maintained at 300 K using the velocity-rescaling thermostat [24], while pressure was controlled at 1 bar.

Long-range electrostatic interactions were treated using the Particle Mesh Ewald (PME) method [25]. Covalent bonds involving hydrogen atoms were constrained using the LINCS algorithm [26], allowing the use of a 2 fs integration timestep.

Production simulations were then performed under periodic boundary conditions for 100 ns per replica.

### 2.4. Trajectory Analysis

Trajectory analyses were carried out using standard GROMACS analysis tools together with custom scripts. All descriptors were averaged across the three independent simulations.

#### 2.4.1. Global Structural Stability (RMSD)

The root mean square deviation (RMSD) of protein backbone atoms was calculated relative to the minimized starting structure after least-squares fitting to remove global translation and rotation. RMSD analysis is widely used to evaluate structural stability and convergence during molecular dynamics simulations [12,15].

#### 2.4.2. Residue-Level Flexibility (RMSF)

Root mean square fluctuation (RMSF) values were calculated for each residue over the equilibrated portion of the trajectories to quantify residue-level flexibility and identify ligand-dependent modulation of protein dynamics [15].

#### 2.4.3. Protein–Ligand Distance Monitoring

Protein–ligand distances were monitored during the simulations using both center-of-mass distances and minimum heavy-atom distances between ligand and protein residues. These descriptors provide information on the persistence and stability of ligand association within the binding region.

#### 2.4.4. Hydrogen Bond and Contact Analysis

Ligand–protein hydrogen bonds were identified using geometric criteria based on donor–acceptor distance (<3.5 Å) and hydrogen–donor–acceptor angle (>120°). Hydrogen bond occupancy was calculated as the percentage of simulation frames in which a given interaction was present. In addition, ligand–protein contact analysis was performed to identify residues most frequently involved in ligand stabilization.

#### 2.4.5. Secondary Structure Analysis

Secondary structure evolution during the simulations was evaluated using the DSSP algorithm (Define Secondary Structure of Proteins) [27], which assigns secondary structure elements based on hydrogen-bonding patterns and backbone geometry.

### 2.5. Statistical Analysis

Statistical comparisons between the protein–myo-inositol and protein–D-chiro-inositol complexes were performed using the values obtained from three independent molecular dynamics simulations for each system. For each descriptor—including backbone RMSD, RMSF within the binding region, ligand–protein distances, hydrogen bond counts, and secondary structure content—mean values and standard deviations were calculated across the replicate trajectories. Statistical significance between the two complexes was evaluated using a two-tailed Student’s *t*-test. Differences were considered statistically significant when *p* < 0.05.

## 3. Results

### 3.1. Docking-Derived Binding Modes

Molecular docking simulations were first performed to generate plausible binding configurations of myo-inositol and D-chiro-inositol within the recognition region of the protein.

The docking results indicate that both stereoisomers occupy the same binding region of the protein surface. The binding pocket is characterized by several polar residues capable of forming hydrogen bonds with the multiple hydroxyl groups present in the inositol ring. This polar environment is therefore well suited to accommodate highly hydrophilic ligands such as inositols.

Although the two stereoisomers bind within the same region, the docking poses reveal subtle differences in ligand orientation relative to the surrounding residues. In particular, the orientation of hydroxyl groups with respect to hydrogen bond donors and acceptors differs between myo-inositol and D-chiro-inositol. These geometrical differences may influence the interaction patterns and the stability of ligand–protein contacts during subsequent molecular dynamics simulations.

**Figure 1:**
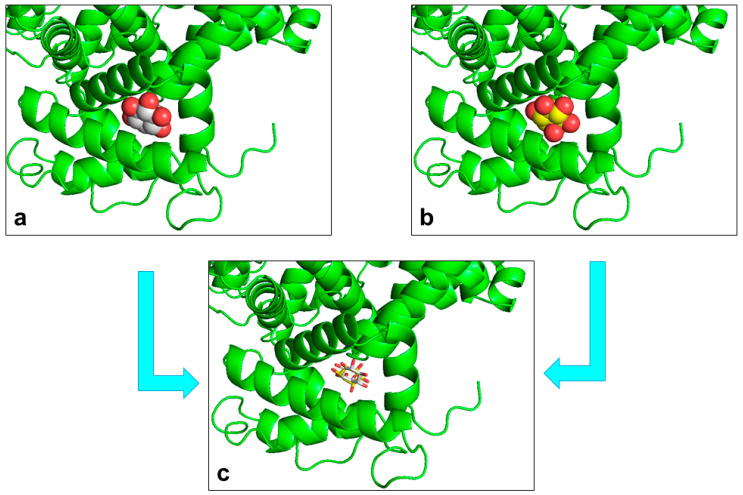
Molecular docking analysis of inositol isomers with bovine serum albumin (BSA). (**a**) Predicted binding mode of myo-inositol within the BSA binding pocket, highlighting key intermolecular interactions stabilizing the complex; the ligand is represented in sphere model. (**b**) Docking pose of D-chiro-inositol in BSA, showing a comparable orientation and interaction pattern; the ligand is represented in sphere model. (**c**) Superposition of myo-inositol and D-chiro-inositol binding sites, demonstrating the overlap and shared binding region within BSA, suggesting a common recognition site for both stereoisomers; ligands are represented in stick model.

Representative docking poses of the two stereoisomers are shown in Figure 1, highlighting the residues involved in initial ligand recognition.

### 3.2. Global Structural Stability of the Complexes

To evaluate the structural stability of the complexes, all-atom molecular dynamics simulations were performed for 100 ns and repeated in triplicate for each system.

**Figure 2:**
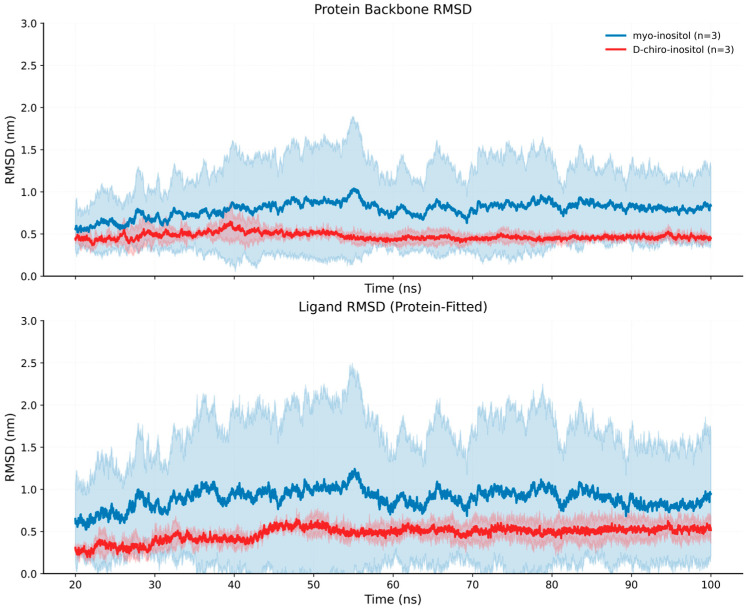
Root mean square deviation (RMSD) profiles of the protein–ligand complexes during molecular dynamics simulations. Backbone RMSD values of the protein (left axis) and RMSD values of the ligand after fitting on the protein backbone (right axis) are shown as a function of simulation time. Shaded regions represent the standard deviation calculated from three independent 100 ns simulations for each complex. The plots illustrate the overall structural stability of the protein–myo-inositol and protein–D-chiro-inositol complexes throughout the trajectories.

The backbone RMSD profiles of the protein during the simulations are shown in Figure 2. In both complexes, the RMSD rapidly increases during the initial phase of the trajectory and subsequently stabilizes, indicating that the systems reach structural equilibrium early in the simulation.

Although both systems remain globally stable, the RMSD distributions reveal subtle differences between the two complexes. The protein–D-chiro-inositol complex displays slightly lower RMSD values and narrower fluctuations compared to the protein–myo-inositol system. Conversely, the myo-inositol complex exhibits somewhat larger conformational excursions while maintaining an overall stable fold. Notably, this difference reflects global conformational variability of the protein scaffold and does not contradict the increased stabilization observed locally at the binding region in the presence of myo-inositol.

Ligand RMSD values calculated after fitting the trajectories to the protein backbone are also reported in Figure 2, providing an indication of the positional stability of the ligand within the binding region throughout the simulation.

Overall, these results indicate that both stereoisomers remain associated with the protein during the entire simulation time, while inducing slightly different conformational responses in the protein scaffold.

This apparent discrepancy between global RMSD and local stabilization suggests that increased global conformational variability does not necessarily reflect reduced ligand association, but may instead indicate differences in protein conformational sampling induced by the two stereoisomers.

### 3.3. Residue-Level Flexibility Analysis

To investigate the influence of ligand binding on local protein dynamics, residue-level flexibility was analyzed through root mean square fluctuation (RMSF) calculations.

**Figure 3:**
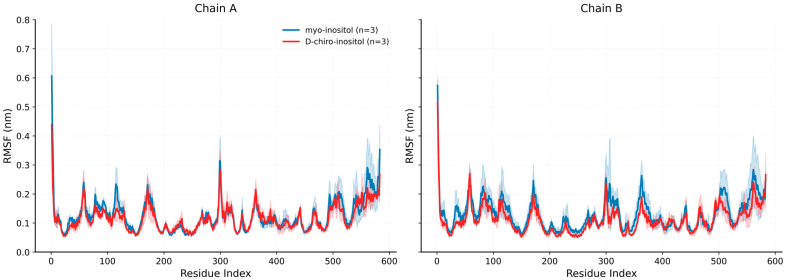
Residue-level flexibility profiles obtained from root mean square fluctuation (RMSF) analysis. RMSF values calculated for each residue of the protein during molecular dynamics simulations over equilibrated trajectories (post-20 ns). Separate plots are shown for chain (**A**) and chain (**B**) of the protein. Solid lines represent the mean RMSF values obtained from three independent simulations of the myo-inositol and D-chiro-inositol complexes, while the shaded areas indicate the standard deviation among replicates. Regions of increased fluctuation correspond primarily to loop and terminal regions.

The RMSF profiles averaged over the three independent simulations are reported in Figure 3, where the results are shown separately for chain A and chain B of the protein. In general, the flexibility patterns are similar in the presence of the two ligands. Higher fluctuations are primarily observed in loop regions and terminal segments, while structured regions such as α-helices exhibit lower mobility.

To highlight differences between the two systems, the differential RMSF was calculated as ΔRMSF = RMSF_myo − RMSF_D-chiro. The resulting profile is shown in Figure S1. Positive values correspond to residues displaying greater flexibility in the myo-inositol complex, whereas negative values indicate regions where D-chiro-inositol induces higher mobility.

The ΔRMSF analysis reveals localized differences in residue flexibility across the protein structure, suggesting that the two stereoisomers may modulate local protein dynamics differently.

### 3.4. Ligand–Protein Distance and Contact Analysis

The stability of ligand association with the protein was further investigated by monitoring ligand–protein distances and the number of atomic contacts throughout the simulations.

**Figure 4:**
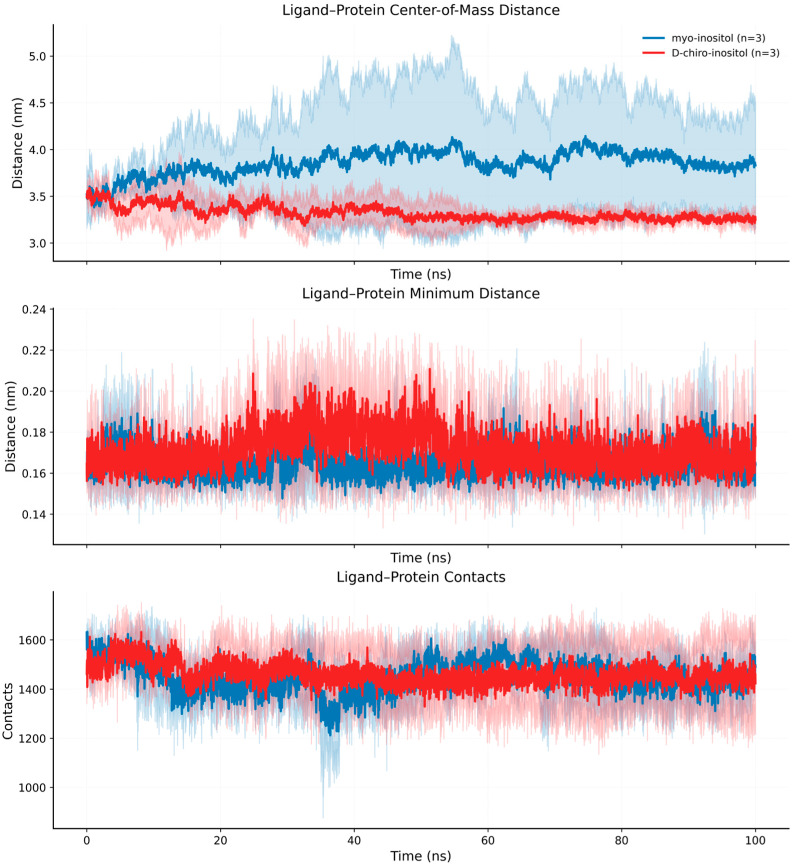
Time evolution of protein–ligand distances and contact patterns during molecular dynamics simulations. Top panels: center-of-mass distance between ligand and protein as a function of simulation time for the two complexes. Middle panels: minimum heavy-atom distance between ligand and protein. Bottom panels: number of atomic contacts between ligand and protein residues during the trajectories. Shaded regions represent the standard deviation across three independent simulations. These descriptors provide information on the stability and persistence of ligand association within the binding region.

The time evolution of the center-of-mass distance between the ligand and protein is shown in Figure 4, together with the minimum heavy-atom distance between ligand and protein residues. Both ligands remain within the binding region during the simulations, with distance values fluctuating around stable average levels.

Interestingly, the myo-inositol complex exhibits a slightly larger average center-of-mass distance compared to the D-chiro-inositol complex. However, the minimum atom–atom distances remain comparable for the two systems, indicating that direct interactions between ligand and protein residues are maintained throughout the trajectories.

The number of ligand–protein contacts over time is also reported in Figure 4, showing relatively stable interaction patterns for both complexes.

**Figure 5:**
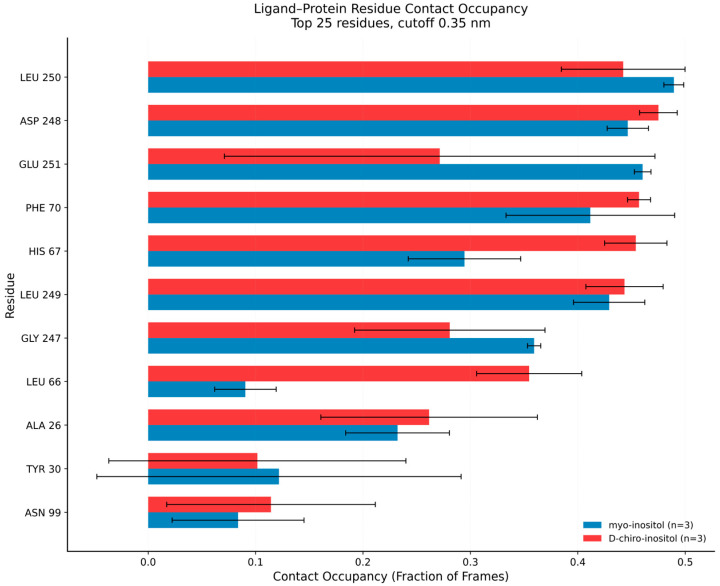
Contact occupancy of the most frequent protein–ligand interactions during the simulations over equilibrated trajectories (post-20 ns). Bar plot showing the occupancy of the most persistent contacts between ligand and protein residues across the trajectories. Contact occupancy represents the percentage of simulation frames in which a given ligand–residue interaction is present. The analysis highlights residues contributing most frequently to ligand stabilization within the binding region.

To further characterize interaction persistence, the occupancy of the most frequent ligand–protein contacts was calculated and is summarized in Figure 5. The analysis highlights several residues—including Tyr30, Gly247, Asp248, Leu249, Leu250, and Glu251—as key contributors to ligand stabilization. These residues exhibit high contact occupancy throughout the trajectories, suggesting their involvement in a relatively persistent interaction network within the binding region. Such contacts likely play an important role in maintaining ligand positioning and mediating the differences observed between the two stereoisomeric complexes.

### 3.5. Secondary Structure Evolution

To evaluate whether ligand binding induces structural rearrangements in the protein, secondary structure evolution was analyzed throughout the simulations using the DSSP algorithm.

**Figure 6:**
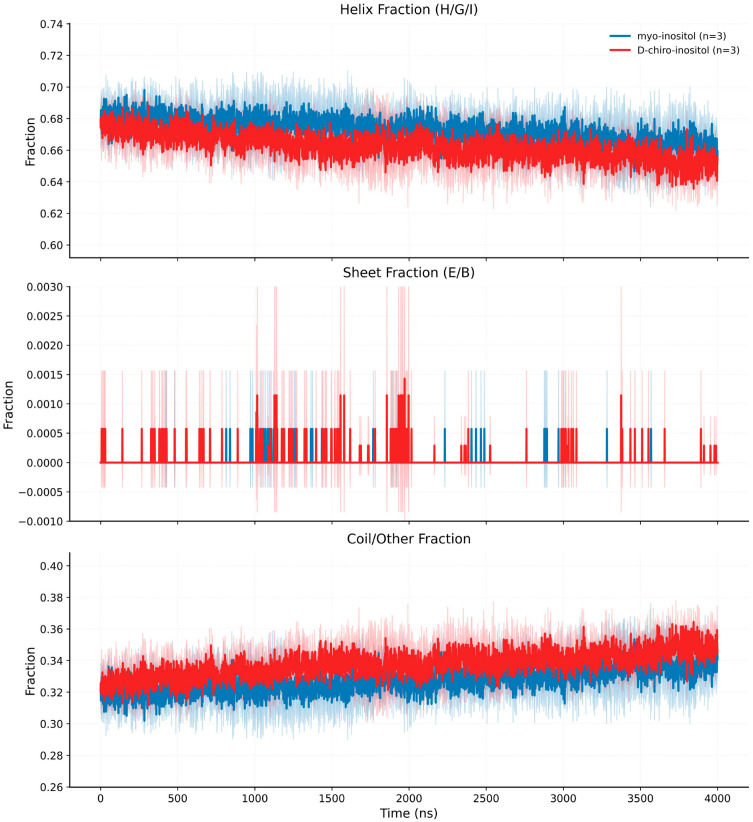
Secondary structure evolution of the protein during molecular dynamics simulations over equilibrated trajectories (post-20 ns). Fraction of α-helix, β-sheet, and coil/other secondary structure elements as a function of simulation time for the myo-inositol and D-chiro-inositol complexes. Secondary structure assignments were calculated using DSSP analysis. Solid lines represent the mean values obtained from three independent simulations, while the shaded regions indicate the standard deviation among replicates. The plots show that the overall secondary structure composition remains largely stable during the simulations.

The fractions of α-helix, β-sheet, and coil/other secondary structure elements as a function of simulation time are shown in Figure 6 for both complexes.

The results indicate that the overall secondary structure composition remains largely stable during the simulations. The α-helical content represents the dominant structural component, while β-sheet structures appear only sporadically. Coil and loop regions account for the remaining fraction of the protein structure.

These observations suggest that ligand binding does not induce large-scale structural rearrangements in the protein scaffold over the simulated timescale.

To provide a quantitative comparison between the two complexes, key dynamic descriptors extracted from the trajectories were summarized and statistically compared. These parameters include backbone RMSD, RMSF within the binding region, average ligand–protein distance, number and occupancy of hydrogen bonds, and the fraction of ordered secondary structure elements. The resulting values, averaged across the three independent simulations for each system, are reported in Table 1. Overall, the results suggest trends consistent with a more persistent interaction pattern in the myo-inositol complex compared with the D-chiro-inositol system, although several descriptors did not reach statistical significance.

To further support the dynamic interpretation of ligand recognition, Molecular Mechanics/Poisson–Boltzmann Surface Area MM-PBSA binding free energy calculations were performed on the equilibrated portions of the MD trajectories. The myo-inositol complex showed a more favorable average binding free energy than the D-chiro-inositol complex (−149.95 ± 3.35 versus −126.19 ± 41.49, respectively). However, this difference did not reach statistical significance, mainly due to the high variability observed among D-chiro-inositol replicas. Therefore, MM-PBSA results should be interpreted as supporting a trend toward more favorable myo-inositol binding rather than as definitive evidence of a statistically distinct binding affinity. This trend is consistent with the experimentally observed stereoselective response and with the MD-derived differences in interaction persistence and local protein dynamics.

## 4. Discussion

These findings extend our previous observations [11], where docking analysis suggested comparable binding regions and interaction patterns for both stereoisomers. While docking provided a static view of ligand recognition, the present molecular dynamics results reveal that stereoselectivity arises from differences in interaction persistence and protein dynamic response.

The present computational study provides a molecular-level perspective on the interaction between two stereoisomeric ligands, myo-inositol and D-chiro-inositol, and the protein recognition element employed in the stereoselective biosensor system. Although the two molecules differ only in the stereochemical orientation of a single hydroxyl group, the simulations reveal that this subtle structural variation leads to measurable differences in ligand orientation and protein conformational behavior.

Docking simulations indicate that both stereoisomers bind within the same recognition region of the protein. This observation suggests that the stereoselective response observed experimentally cannot be explained by the presence of distinct binding pockets. Instead, the two ligands adopt slightly different orientations within the same polar environment, leading to variations in hydrogen bond geometry and interaction patterns. In highly hydrophilic systems dominated by hydrogen bonding, even small stereochemical differences may significantly influence interaction cooperativity and binding geometry [7]. In addition to direct ligand–protein contacts, solvent-mediated effects are likely to contribute significantly to stereoselective recognition in this highly hydrophilic system. Because both inositol stereoisomers contain multiple hydroxyl groups exposed to the aqueous environment, competition between ligand–protein and ligand–water hydrogen bonding may strongly influence interaction persistence and local conformational behavior. Small differences in hydroxyl orientation can modify the geometry, lifetime, and cooperativity of hydrogen bond networks within the binding region, thereby affecting transient interaction patterns and protein dynamic fluctuations during the simulations. These observations further support the view that stereoselective recognition in this biosensor system emerges from cumulative dynamic and solvent-mediated effects rather than from large differences in static affinity alone.

Molecular dynamics simulations confirm that the docking-derived complexes remain stable during the simulated timescale. Both ligands remain associated with the protein throughout the trajectories, indicating that the predicted binding poses correspond to stable interaction configurations. However, the RMSD analysis suggests that the two complexes explore somewhat different conformational landscapes. While both systems maintain overall structural stability, the protein–myo-inositol complex exhibits slightly larger conformational fluctuations compared to the D-chiro-inositol complex. These differences likely reflect alternative interaction geometries rather than substantial differences in binding affinity.

Residue-level flexibility analysis provides further insight into ligand-dependent modulation of protein dynamics. Although the overall RMSF profiles are broadly similar, localized differences are observed across several regions of the protein. The ΔRMSF analysis highlights that the influence of the two stereoisomers on protein flexibility is spatially heterogeneous. Such ligand-dependent modulation of local dynamics is commonly observed in protein–ligand systems and reflects the coupling between ligand binding and the intrinsic conformational landscape of proteins [16,17].

The analysis of ligand–protein distances and contact occupancy further illustrates the distinct interaction modes adopted by the two stereoisomers. Interestingly, the myo-inositol complex exhibits a slightly larger average center-of-mass distance from the protein compared to D-chiro-inositol while maintaining comparable minimum atomic distances and a similar number of ligand–protein contacts. This observation suggests that the two ligands interact with the recognition region through different geometric arrangements rather than through simple proximity effects. The contact occupancy analysis indicates that several residues—including Tyr30, Gly247, Asp248, Leu249, Leu250, and Glu251—are frequently involved in ligand stabilization.

Secondary structure analysis indicates that ligand binding does not induce major structural rearrangements in the protein scaffold. The relative fractions of α-helical, β-sheet, and coil elements remain largely constant throughout the simulations. This behavior is consistent with many protein–ligand systems in which binding events primarily influence local dynamics rather than inducing large-scale conformational transitions [28].

From the perspective of biosensor function, these observations are particularly relevant. Electrochemical biosensors based on immobilized proteins detect target molecules through subtle changes in interfacial properties, including charge distribution, molecular organization, and dynamic fluctuations at the electrode surface. Ligand binding may therefore influence the structural organization and dynamic behavior of the protein layer, ultimately modulating electron-transfer processes and interfacial impedance [29]. From a bioreceptor engineering perspective, these results suggest that even subtle ligand-dependent changes in protein conformational dynamics and interaction persistence may contribute to modulation of the electrochemical response at the electrode interface. Even relatively small ligand-dependent differences in local conformational dynamics may become amplified at the electrochemical interface through collective effects within the immobilized protein layer, thereby contributing to measurable differences in signal response despite the absence of major structural rearrangements. Computational characterization of these dynamic effects may therefore support the rational optimization of protein-based sensing platforms for stereochemically similar targets.

Taken together, the results of this study suggest that the discrimination between myo-inositol and D-chiro-inositol arises from subtle differences in ligand–protein interaction geometry and in the dynamic response of the protein scaffold. Rather than involving distinct binding sites or large structural rearrangements, stereoselective recognition appears to emerge from differences in interaction networks and local conformational fluctuations. MM-PBSA calculations further support this interpretation by showing a trend toward more favorable binding free energy for myo-inositol, although the variability observed among replicas prevented statistical significance. These findings therefore support a dynamic interpretation of stereoselective recognition rather than indicating large differences in static affinity.

More broadly, these findings highlight the importance of considering conformational dynamics when investigating the molecular basis of selectivity in protein-based biosensors. The integration of molecular docking and molecular dynamics simulations therefore represents a valuable strategy for elucidating the mechanisms underlying biosensor recognition processes at the molecular level.

## 5. Conclusions

This study provides a computational investigation of the interaction between two stereoisomeric metabolites, myo-inositol and D-chiro-inositol, and the protein recognition element employed in a stereoselective electrochemical biosensor. Although the two molecules differ only in the stereochemical orientation of a single hydroxyl group, molecular docking and molecular dynamics simulations reveal that this subtle structural variation influences ligand orientation within the binding region and the dynamic response of the protein scaffold.

Docking results indicate that both stereoisomers occupy the same recognition region of the protein, suggesting that stereoselective sensing does not arise from the presence of distinct binding pockets. Instead, the two ligands adopt slightly different orientations within the binding site, resulting in variations in hydrogen bond geometry and residue-level interactions. Molecular dynamics simulations further demonstrate that both complexes remain stable throughout the simulated timescale while displaying distinct patterns of conformational fluctuations and ligand–protein contact persistence.

Residue-level flexibility analysis, ligand–protein distance monitoring, and contact occupancy calculations collectively suggest that the two stereoisomers interact with the protein through different geometric arrangements that modulate local protein dynamics without inducing major structural rearrangements. These subtle differences in interaction networks may contribute to the differential electrochemical response observed experimentally for the stereoselective myo-inositol biosensor.

Overall, this work highlights the importance of considering conformational dynamics when investigating the molecular determinants of selectivity in protein-based biosensors. The combined use of molecular docking and molecular dynamics simulations provides a useful framework for elucidating stereochemical recognition mechanisms and can support the rational design and interpretation of biosensing platforms targeting structurally similar metabolites.

## Figures and Tables

**Table 1:** Comparative Dynamic and Energetic Parameters Derived from Molecular Dynamics Simulations; Summary of molecular dynamics-derived structural and interaction parameters for complexes of bovine serum albumin (BSA) with myo-inositol and D-chiro-inositol. Reported values represent mean ± standard deviation calculated over equilibrated trajectories (post-20 ns). Backbone RMSD and ligand-fit RMSD describe global protein stability, while RMSF values (Cα and core atoms) quantify residue-level flexibility within the binding region and its extended environment. Ligand–protein center-of-mass (COM) distance and minimum distance (mindist) evaluate spatial proximity, whereas contact counts reflect interaction persistence. Statistical significance between systems was assessed using Student’s *t*-test, with corresponding *p*-values reported.

Parameter	Myo-Inositol	D-Chiro-Inositol	*p*-Value
Backbone RMSD (nm)	0.787 ± 0.491	0.467 ± 0.025	0.323
Ligand fit Backbone RMSD (nm)	0.887 ± 0.802	0.470 ± 0.053	0.420
RMSF (CA and Core) (Binding Region, nm)	0.083 ± 0.007	0.067 ± 0.002	0.053
RMSF (CA and Core) (Binding Region Extended, nm)	0.093 ± 0.007	0.074 ± 0.001	0.051
Ligand–Protein COM Distance (Å)	3.892 ± 0.660	3.296 ± 0.063	0.194
Ligand–Protein mindist Distance (Å)	0.165 ± 0.001	0.173 ± 0.008	0.145
Ligand–Protein Contact (count)	1439.7 ± 47.5	1452.1 ± 118.6	0.875
MM-PBSA Binding Free Energy (kJ/mol)	−149.9 ± 3.4	−126.2 ± 41.5	0.379

## Data Availability

The data presented in this study are contained within the article. Further inquiries can be directed to the corresponding author.

## Supplementary Materials

The following supporting information can be downloaded at: https://www.mdpi.com/article/10.3390/s26123765/s1, Figure S1: Differential residue flexibility between the two complexes (ΔRMSF analysis) over equilibrated trajectories (post-20 ns). ΔRMSF values calculated as the difference between RMSF values of the myo-inositol and D-chiro-inositol complexes (ΔRMSF = RMSF_myo − RMSF_D-chiro). Solid lines represent the mean values obtained from three independent simulations, while the shaded regions indicate the standard deviation among replicates. Positive values indicate residues exhibiting greater flexibility in the myo-inositol complex, whereas negative values correspond to regions where D-chiro-inositol induces higher mobility.
