# Supplementary material for: Molecular Dynamics Analysis of the Stereoselective Recognition of Myo-Inositol and D-Chiro-Inositol in a Protein-Based Biosensor
Source: Sensors (Basel). 2026 Jun 12;26(12):3765. doi: 10.3390/s26123765 (PMC13307042; doi:10.3390/s26123765)
Supplement: Supplementary file 1 [file sensors-26-03765-s001.zip › sensors-4291579-Supplementary.pdf]

sensors

MDPI

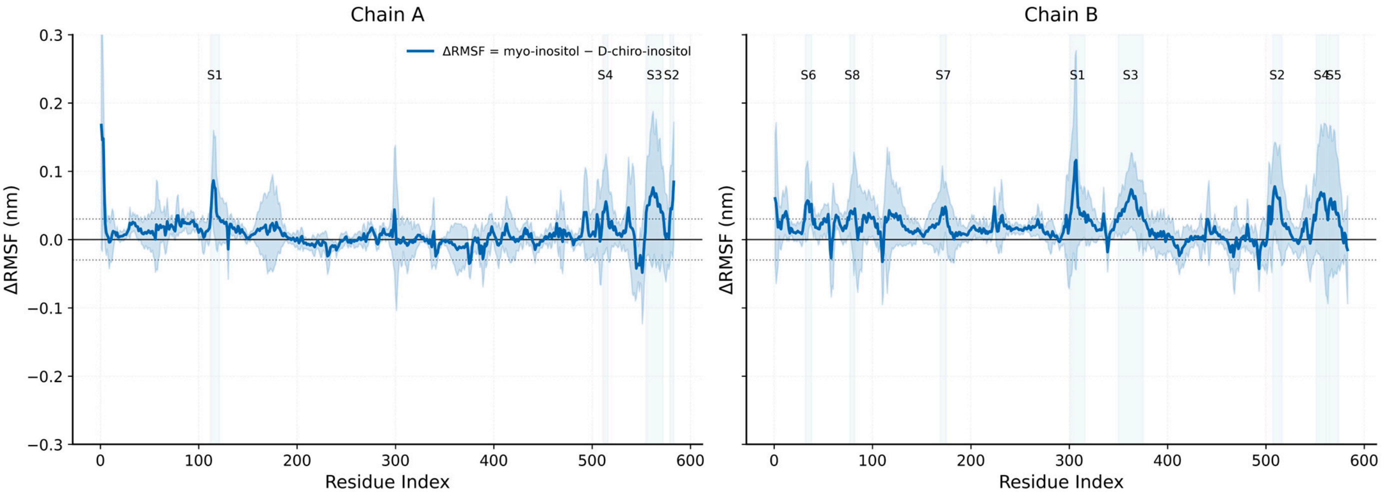

Figure S1. Differential residue flexibility between the two complexes (ΔRMSF analysis) over equilibrated trajectories (post-20 ns). ΔRMSF values calculated as the difference between RMSF values of the myo-inositol and D-chiro-inositol complexes (ΔRMSF = RMSF_myo − RMSF_D-chiro). Solid lines represent the mean values obtained from three independent simulations, while the shaded regions indicate the standard deviation among replicates. Positive values indicate residues exhibiting greater flexibility in the myo-inositol complex, whereas negative values correspond to regions where D-chiro-inositol induces higher mobility.
